# Integrating Human Health into Environmental Impact Assessment: An Unrealized Opportunity for Environmental Health and Justice

**DOI:** 10.1289/ehp.11132

**Published:** 2008-04-16

**Authors:** Rajiv Bhatia, Aaron Wernham

**Affiliations:** 1 San Francisco Department of Public Health, San Francisco, California, USA; 2 Alaska Inter-Tribal Council, Fairbanks, Alaska, USA

**Keywords:** environmental health, environmental impact assessment, environmental justice, health determinants, health disparities, health impact assessment, public policy, social justice

## Abstract

**Objectives:**

The National Environmental Policy Act and related state laws require many public agencies to analyze and disclose potentially significant environmental effects of agency actions, including effects on human health. In this paper we review the purpose and procedures of environmental impact assessment (EIA), existing regulatory requirements for health effects analysis, and potential barriers to and opportunities for improving integration of human health concerns within the EIA process.

**Data sources:**

We use statutes, regulations, guidelines, court opinions, and empirical research on EIA along with recent case examples of integrated health impact assessment (HIA)/EIA at both the state and federal level.

**Data synthesis:**

We extract lessons and recommendations for integrated HIA/EIA practice from both existing practices as well as case studies.

**Conclusions:**

The case studies demonstrate the adequacy, scope, and power of existing statutory requirements for health analysis within EIA. The following support the success of integrated HIA/EIA: a proponent recognizing EIA as an available regulatory strategy for public health; the openness of the agency conducting the EIA; involvement of public health institutions; and complementary objectives among community stakeholders and health practitioners. We recommend greater collaboration among institutions responsible for EIA, public health institutions, and affected stakeholders along with guidance, resources, and training for integrated HIA/EIA practice.

A major achievement of the emerging environmental movement in the United States, the National Environmental Policy Act of 1969 (NEPA) established a foundation for environmental policy in the United States. This far-reaching legislation required that any “major federal action significantly affecting the quality of the human environment” must undergo an evaluation and public disclosure of its environmental effects (NEPA 1969). To accomplish this mandate, NEPA institutionalized the now-ubiquitous environmental impact statement (EIS).

For almost four decades, NEPA has been a powerful and influential tool for environmental protection [Council on Environmental Quality (CEQ) 1997a]. At least 19 states or territories have now enacted statutes requiring some form of environmental impact assessment (EIA). NEPA applies to a striking range of activity including, for example, highways and other transit projects and programs, natural resource leasing and extraction, industrial farming and policies governing genetically modified crops, and large-scale urban redevelopment projects. Every executive branch federal agency uses the NEPA process. More than 500 federal programs undergo an EIS annually, and thousands more are evaluated using a similar but less-detailed process termed “environmental assessment.” State statutes such as the California Environmental Quality Act (CEQA) capture a still wider range of activity, often including smaller-scale development projects as well as state programs for natural resources management and public infrastructure development.

Projects, policies, and programs subject to EIA influence not only environmental quality but also industry and employment patterns, regional economies, the built environment, social organization, and culture—important determinants of health and well-being. Viewed collectively, the range of activity subject to state or federal EIA exerts a profound influence on health in communities across the United States.

The protection of human health and welfare figures prominently in the objectives and regulations of NEPA (CEQ 1978; NEPA 1969). In practice, however, the consideration of health within EIA is both rare and narrowly focused on toxic exposures; a comprehensive and systematic approach to human health impacts in EIA practice has not evolved (Davies and Sadler 1997; National Research Council 2003; Steinemann 2000).

The inattention to health in EIA practice stands in contrast to the interdependence among environmental change, societal conditions, and human health. Environmental change—including issues as diverse as global warming, deforestation, fisheries loss, and suburban sprawl—is now seen as a priority challenge to public health. The World Health Organization (WHO), for example, recently estimated that over 25% of the burden of human illness worldwide can be attributed to modifiable environmental conditions (Frumkin 2004; Pruss-Ustun and Corvalan 2006), and evidence linking social conditions such as employment, transportation, housing, food resources, social hierarchy, economic disparity, and social capital to health outcomes continues to grow in strength and depth (Marmot and Wilkinson 2006).

In 1986, the Ottawa Charter on Health Promotion urged policy makers in all sectors to “be aware of the health consequences of their decisions and to accept their responsibilities for health” (WHO 1986). It is increasingly clear that effective health strategies in the 21st century will require both the involvement of all public sectors and attention to the diverse social, economic, and environmental forces that shape community health (Hanna and Coussens 2001).

Here we draw on the experience of public health practitioners in two settings to demonstrate that the statutory and procedural requirements of EIA provide a powerful and underutilized mechanism to institutionalize a holistic, cross-sectoral approach to addressing health in public policy. We review EIA requirements under two laws, NEPA and CEQA, and consider why health has not become a central focus of modern EIA practice. We then discuss the emerging practice of health impact assessment (HIA) as a catalyst for integrating health considerations into EIA practice and describe early U.S. examples of integrated HIA/EIA. Finally, we discuss lessons learned from the experiences of the authors and steps toward a more active role for public health in shaping the policies and decisions made using EIA.

## EIA Statutes: Policy, Process, and Stakeholder Participation

In contrast to laws that promulgate specific regulatory standards, such as the Clean Air Act (1990), NEPA established EIA based on the premise that a full, public accounting of the potential environmental effects of public decisions would promote environmentally responsible public policy and regulatory decisions (Karkkainen 2002). The EIA is intended as an informational document that allows the public and decision makers to understand the potential impacts of a proposal. Although specific requirements and terminology vary somewhat depending on the governing statute, most EIA statutes share the same basic requirements for a comprehensive assessment of potential environmental effects and for public participation. In this review we focus on the requirements of NEPA and CEQA to illustrate general features common to most EIA statutes.

Both NEPA and CEQA are triggered when an agency decision is likely to impact the physical environment, either directly or indirectly. Under both laws, a less-detailed, screening-level assessment may be used to determine the need for and scope of a full EIA (CCR 2007 §15063; CEQ 1978 §1508.9). Both statutes require that the EIA contain *a*) a description of the environment affected by the proposed action; *b*) an assessment of the direct and indirect environmental impacts effects of the proposed action; and *c*) an analysis of reasonable alternatives to the proposed action, generally including a “no-action alternative” and various iterations of the proposed activity (Yost 2003). The development and analysis of alternatives is intended to provide options that maximize benefits while minimizing potential harms. Both statutes also encourage agencies to identify, consider, and adopt specific mitigation measures, if available, to minimize any impacts identified. Finally, in cases where the adverse impacts of the proposed action appear too great, an agency may select the no-action alternative or deny approval for the proposal.

Under NEPA, the agency must adopt an enforceable Record of Decision, which specifies what activities will be permitted, what mitigation measures are required, and how the EIA informed these decisions (CEQ 1978 §1505.2.) Under CEQA, if the EIA identifies significant environmental impacts, the lead agency must adopt findings identifying the required mitigations incorporated into the project. Certification of an EIA with unmitigated significant environmental impacts requires a formal Statement of Overriding Considerations that documents the project’s compelling benefits and the specific economic, legal, social, or technologic barriers that make mitigations or alternatives infeasible.

Both NEPA and CEQA also have strong provisions for public input, with mandates for public comment periods during which stakeholders—including affected communities, public agencies, and project proponents—may submit input on the scope and adequacy of the EIA analysis. Both add force to public input by requiring that agencies respond to all substantive comments, accounting for the input either by modifying the EIA or by justifying the original analysis.

NEPA also mandates the responsible, or lead, agency to solicit participation by state, local, and tribal governments and agencies with legal jurisdiction or relevant expertise [CEQ 1978 § 4331(a), §4332(2)]. According to the White House CEQ—the body created by NEPA to oversee its implementation—“The benefits of enhanced cooperating agency participation in the preparation of NEPA analyses include: disclosing relevant information early in the analytical process; applying available technical expertise and staff support; avoiding duplication with other federal, state, tribal and local procedures; and establishing a mechanism for addressing intergovernmental issues” (Connaughton 2002). Federal agencies with legal jurisdiction must be cooperating agencies (CEQ 1978 §1501.6). Cooperating agency status allows an agency, community, or tribe considerable opportunity to influence an EIA by participating in developing the scope, analytic approach, and selection of alternatives, as well as by drafting sections of the EIA itself.

Despite over three decades of practice, some criticize the process of EIA as a bureaucratic hurdle that creates costly and duplicative information requirements, unnecessary procedural delays, and opportunities for special interest litigation (Black 2004; Karkkainen 2002). Despite these criticisms, however, there is considerable evidence that EIA statutes contribute to environmental protection and offer a process through which impacted communities can ensure that regulatory decisions are responsive to their concerns (Canter and Clark 1997; CEQ 1997a). Furthermore, EIA also offers avenues for a legal remedy if stakeholders are not satisfied that the process addresses their substantive concerns. The findings of an EIA and, at times, the associated public outcry, can also influence ultimate decisions regarding the fate of the project (Douglas et al. 2005).

A survey of NEPA scholars and practitioners conducted 25 years after its enactment found both strengths and room for improvement (Canter and Clark 1997). Responses suggested that important strengths of NEPA include encouraging agencies to identify, study, and acknowledge potential environmental consequences, and to consider these consequences in their management decisions. At the same time, the survey revealed deficiencies, including the need for *a*) monitoring of impacts and mitigation efficacy, *b*) methodologic approaches to cumulative impact assessment, *c*) earlier consideration of environmental impacts in the planning process, and *d*) a more integrated consideration of biophysical and social impacts.

## Health in U.S. EIA Regulations and Practice

NEPA explicitly recognizes the interdependence of environmental quality and human health. One of the stated purposes of NEPA is “to promote efforts which will prevent or eliminate damage to the environment and biosphere and stimulate the health and welfare of man” (NEPA 1969 §4321). NEPA charged the federal government “to use all practicable means, consistent with other essential considerations of national policy” to “assure for all Americans safe, healthful, productive and aesthetically and culturally pleasing surroundings” (NEPA 1969 §4331).

The regulations for implementing NEPA define the human environment as “the natural and physical environment and the relationship of people with that environment” (CEQ 1978 §1508.14). The regulations define effects subject to analysis as those that are “ecological, aesthetic, historic, cultural, economic, social, or health, whether direct, indirect, or cumulative” (CEQ 1978 §1508.8). Furthermore, in determining whether an effect is significant, an agency must evaluate the “degree to which the proposed action affects public health or safety” (CEQ 1978 §1508.27).

The term “human environment” expressed the intent of Congress for NEPA to promote public policy attentive to the interrelated needs of human well-being and environmental integrity. Senator Henry Jackson (1969), the key author of NEPA, expressed this clearly: “When we speak of the environment, basically, we are talking about the relationship between man and these physical and biological and social forces that impact upon him. A public policy for the environment basically is not a public policy for those things out there. It is a policy for people.”

Executive Order 12898, rooted in the Equal Protection Clause of the U.S. Constitution, provides a more recent impetus for health effects analysis in EIA, requiring that “each Federal agency make achieving environmental justice part of its mission by identifying and addressing, as appropriate, disproportionately high adverse human health and environmental effects of its programs, policies, and activities on minority populations and low income populations” (Clinton 1994). The CEQ guidance on implementing Executive Order 12898 directs federal agencies to consider “ecological, cultural, human health, economic, or social impacts on minority communities, low-income communities, or Indian tribes when those impacts are interrelated to impacts on the natural or physical environment” (CEQ 1997b). In an example of the use of this guidance, the Nuclear Regulatory Commission denied the license for a nuclear fuel enrichment facility near two small, predominantly minority rural communities because the project would have eliminated a road between the two communities (Bass 1998).

Surprisingly little case law has considered requirements for health analysis within EIA under NEPA, perhaps because of the limited health analysis actually conducted within NEPA, and because plaintiffs in NEPA litigation have most commonly represented ecologic concerns. *Metropolitan Edison Co v. People against Nuclear Energy* (PANE) (1983), a case in the Supreme Court, addressed impacts on psychological well-being under NEPA (Bauser 1985; Cole 2004). The court ruled that adverse psychological effects resulting from the perception of risk associated with nuclear power generation were not cognizable under NEPA, because the risk of a risk was too distal a causal connection. This ruling has been the subject of considerable debate (e.g., Bauser 1985 and Jordan 1984). In writing the court’s opinion, however, Chief Justice Rehnquist noted that “all parties agree that effects on human health can be cognizable under NEPA, and that human health may include psychological health.” The authors, along with the former general counsel to the CEQ, believe that the PANE decision spoke primarily to the length of the causal chain as opposed to the place of psychological impacts under NEPA (D. Bear, personal communication).

State-level versions of NEPA vary. However, of the 17 state NEPA-like statutes referenced on the NEPA website (http://www.nepa.gov/nepa/regs/states/states.cfm), 14 contain language that might support the inclusion of health. In California, CEQA requires an environmental impact report (EIR) whenever the environmental effects of a project have the potential to cause substantial adverse effects on human beings, either directly or indirectly (CEQA 2005; CCR 2007 §15065). CEQA regulations also specifically require that an EIR discuss health and safety problems caused by the physical changes (CCR 2007 §15126.2).

In California under CEQA, case law has provided more explicit support for health analysis in EIA. In *Bakersfield Citizens v. City of Bakersfield* (2004), the court found an EIR inadequate because of its failure to analyze the respiratory health impacts caused by the impacts of the projects on air quality. In the court’s opinion, “After reading the EIRs, the public would have no idea of the health consequences that result when more pollutants are added to a non-attainment basin.” In another case, the court found a California Department of Food and Agriculture (CDFA) EIR deficient because it did not adequately evaluate the health impacts of authorizing pesticide use, including impacts on people in nonagricultural areas (*Californians for Alternatives to Toxics v. CDFA* 2005).

Surprisingly, despite the intent and requirements of EIA statues, in practice EIA has not developed a systematic and comprehensive approach to health, either in the United States or internationally (Arquiaga 1994; Cole 2004; Davies and Sadler 1997; Steinemann 2000; Wernham 2007). One review of 42 federal EIAs conducted under NEPA found that more than half contained no mention of health; a minority contained narrow discussions of health risks (usually cancer risk assessments) associated with chemicals or radiation (Steinemann 2000). Commonly, conformity with existing applicable environmental regulations such as the Clean Air Act (1990) serves as a proxy for the health and safety performance of a project, and EIA rarely extends to consider health impacts mediated via changes in the built environment, social determinants, or economic impacts (Davies and Sadler 1997).

Institutional, organizational, and disciplinary factors all help explain the inattention to health within EIA (Rattle and Kwiatkowski 2003). EIA practice evolved primarily in agencies with mandates for environmental management and protection. Currently, neither responsible agencies nor public health officials generally view EIA as an avenue to address health objectives (Noble and Bronson 2006). Assumptions about the appropriate scope of EIA naturally derive from the regulatory mandates of the agencies undertaking EIA and, with time, have become entrenched as precedents (California Department of Transportation 1997; Cole 2004). Thus, currently EIA is largely accomplished by agency staff or by private consultants who lack health expertise. Regulatory agencies rarely request input from health agencies, and there is no established mechanism or expectation for such interaction.

In addition, U.S. EIA statutes do not explicitly describe the kinds of public health effects to be included in an EIA, and to date there is no guidance that specifies the appropriate scope, standards, or methods for analyzing health effects. In the absence of such guidance, the specific health-related requirements of environmental regulatory acts such as the Clean Air Act (1990) and Clean Water Act (1972) often define the scope of health concerns within EIA.

The rigid boundaries among disciplines of knowledge along with semiautonomous development of knowledge in each field have been long-standing obstacles to interdisciplinary thought and practice necessary for integrated impact assessment (Rattle and Kwiatkowski 2003). Relationships between environmental change and human health are emerging, complex, and dynamic, posing challenges to both conceptualizing pathways resulting in human health effects and developing impact assessment methods to assess such effects.

## HIA: A Potential Catalyst for Integrating Health in EIA

HIA describes a diverse set of processes along with a range of qualitative or quantitative methods to identify the health effects of public policy decisions, which, in turn, help to shape policy to promote and protect health (Kemm and Parry 2004; Quigley et al. 2006). HIA is patterned after EIA, with a number of procedural steps accompanied by opportunities for stakeholder involvement (Quigley et al. 2006).

HIA views health holistically, considering not only biophysical health effects, but also broader social, economic, and environmental influences. HIA also explicitly focuses on health benefits and the distribution of health impacts within a population (health equity). Like EIA, HIA strives to anticipate potential impacts for decision makers and to deliver a set of concrete recommendations targeted at minimizing health risks and maximizing benefits.

Broadly, current HIA approaches may be categorized into two groups: *a*) HIA independent of EIA, or voluntary, and *b*) HIA formally integrated with EIA, or regulatory (Cole et al. 2004; Dannenberg et al. 2006). In European Union nations such as England and Sweden, HIA has evolved independent of EIA and is applied in a wide range of public policy decisions not subject to EIA (Kemm and Parry 2004). On the other hand, countries such as Canada and Australia have developed formal guidance for integrating HIA into EIA (EnHealth 2001; Health Canada 2004). Australian HIA guidance explicitly recognizes that the interdisciplinary approach offered by integrated HIA/EIA is desirable and efficient (Wright 2004). At the same time, the Australian experience has shown that integrated HIA/EIA tends to favor quantitative analytic methods, potentially limiting its scope. Canada has had positive though inconsistent success with expanding the traditional EIA approach to include explicit discussions of health determinants (McCaig 2005; Noble and Bronson 2005).

There is debate in the HIA literature regarding the relative benefits of the voluntary and regulatory approaches. In the United States, proponents of the voluntary approach cite EIA’s procedural rigidity, narrow definition of health, strict rules of evidence, adversarial environment, and focus on the physical environment as reasons to develop HIA outside of the EIA process (Cole et al. 2004). Cole and Fielding (2007) argue that “it is far more likely that HIAs linked to EIA will conform to the limits of EIA rather than HIA truly expanding the scope of EIA.” On the other hand, the voluntary HIAs conducted thus far in the United States vary considerably in scope and focus, analytic methods used, influence on the decision-making process, and opportunities for public participation (Dannenberg et al. 2008).

Proponents of integrated HIA/EIA in the United States note that it can use existing statutory requirements for impact assessment and leverage explicit requirements for the mitigation of adverse impacts (Bhatia 2007; Dannenberg et al. 2006; Wernham 2007). Furthermore, the procedural rules and legal levers offered by EIA statutes offer a consistent, substantive opportunity for community engagement in government decision making. Finally, because EIA formally requires the involvement of a range of institutions and disciplines, integrated HIA/EIA provides access to the multidisciplinary expertise required for analysis of complex pathways and a means to engage other public sectors in policy making to protect and promote health.

The cases and discussion below provide further reflection on the value of integrated HIA/EIA in the United States.

## Integrated HIA/EIA: Examples from Practice

The U.S experience with HIA is limited. Dannenberg et al. (2008) documented only 27 completed HIAs between 1999 and 2007, with a range of approaches including independent, voluntary HIAs addressing both EIA-based decisions and policy questions outside the EIA sphere, and formally integrated HIA/EIA. Because the field of HIA is still rapidly evolving in the United States and because evaluation of HIA outcomes has been limited, we believe it is premature to advocate for a single methodologic approach. Below, we present early examples of integrated HIA/EIA from two distinct settings, San Francisco, California, and rural Alaska. These examples suggest that the formal integration of HIA into EIA offers a promising avenue for realizing a truly cross-sectoral approach to a wide range of public policy decisions that impact community health and well-being.

### San Francisco, California: health effects of urban rezoning

Recent experience in San Francisco has demonstrated the ability of a local health department to play a constructive role in EIA and to gain consideration and mitigation of environmental health determinants not usually considered in the EIA process (Bhatia 2007). Traditionally, staff of the Environmental Health Section of the San Francisco Department of Public Health (SFDPH) have sometimes participated in CEQA at the request of the San Francisco Department of City Planning (SFDCP), the lead agency for local CEQA compliance, or the Office of the City Attorney. Health agency roles have included both conducting and reviewing environmental health risk assessments and ensuring that projects comply with local environmental regulations.

Beginning in 2003, public demand emerged for the inclusion of a broader scope of health and social concerns in local EIAs, including the impacts of development on traffic safety; air quality; the adequacy of housing, parks, schools, and community facilities; and displacement of local businesses and low-income populations (Corburn and Bhatia 2007). The SFDPH responded to this demand with a more proactive role in the EIA process.

One of the first SFDPH efforts concerned a proposal to demolish the Trinity Plaza Apartments, comprising 360 rent-controlled units, and rebuild 1,400 new condominiums in its place (Bhatia 2007). In their initial scoping determination, SFDCP officials concluded that the proposal would not have adverse impacts on human populations and housing because the project would contribute a net gain in dwelling units. In contrast, in public testimony, residents and tenant advocates asserted that the city’s determination ignored the human impacts of the proposal—evictions and changes in housing costs. Residents argued that the demolition and displacement of people represented both a direct physical impact on tenants and an indirect impact on their health and well-being.

The SFDPH undertook a brief HIA, synthesizing available data on housing affordability and residential displacement, providing local data on housing conditions, and qualitatively predicting the likely impacts of the demolition and displacement on health. The analysis corroborated community concerns and provided evidence for the likely adverse health consequences of the demolition, including psychological stress, fear, and insecurity due to eviction; crowding or substandard living conditions due to limited affordable replacement housing; food insecurity or hunger due to increased rent burdens; and the loss of supportive social networks due to displacement (SFDPH 2004). Focus groups conducted by the SFDPH provided further corroborating evidence by documenting health effects that were already occurring among tenants threatened with eviction (SFDPH 2005).

The SFDPH input convinced SFDCP officials to revise the required scope of the project’s EIA to include residential displacement and any indirect impacts on health, unless the developer chose to mitigate these effects with a revised plan. Facing the possibility that the EIA would show significant adverse impacts, in tandem with vocal tenant opposition and consideration of legislation for a local moratorium on demolition, the developer agreed to negotiate with tenants and ultimately revised the development proposal to keep 360 of the new units rent-controlled with lifetime leases for existing tenants (Shaw 2005). Because the developer mitigated the impact through project design, a health analysis of displacement was not ultimately required in the project’s EIA. However, in subsequent city planning efforts, EIA has included analysis of residential displacement, and city policies have been developed requiring the replacement of affordable housing lost in the development process (SFDCP 2007a).

Following several similar project-specific efforts, the SFDPH extended its involvement in EIA to a more comprehensive planning process that was seeking to address land use conflicts in four San Francisco neighborhoods—the Eastern Neighborhoods community plans. These plans proposed rezoning land to allow new residential construction near high-volume roadways, existing industrial uses, and freight truck routes, posing a number of important health questions typically not addressed within EIA. For example, analysis of project effects on ambient air quality was routinely included in EIA, yet the traditional approach took compliance with regulatory standards as an adequate proxy for protecting health, ignoring intraurban variation of exposure sources, cumulative effects, and sensitive populations. Similarly, the analysis of noise levels estimated incremental changes but failed to evaluate related impacts of these changes on health. Finally, substantive analysis of the pedestrian safety impacts of development, though supported under CEQA, had not occurred historically.

SFDPH undertook a health analysis of the rezoning plans, focusing on noise, roadway pollution, and pedestrian hazards, and was able to integrate the findings from these analyses directly into the draft EIR for the Eastern Neighborhoods Plans as co-authors (SFDCP 2007b). The draft EIR included new mitigation to require residential projects to analyze roadway pollution and mitigate effects on new residential uses through ventilation systems and building design. Similarly, the draft EIR recognized the significance of impacts of residential–industrial noise conflicts and required a stringent set of new regulations to avoid conflicts associated with mixed-use planning, potentially preventing business displacement. Planners did not create new requirements on development to mitigate pedestrian hazards, but did include polices to reduce traffic within the rezoning plans (SFDCP 2007a).

**Figure 1:**
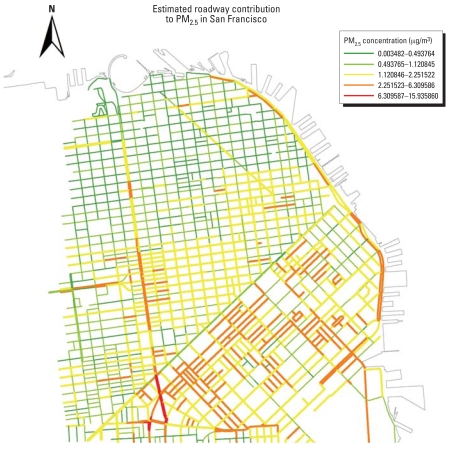
Modeled concentration of PM_2.5_ from traffic sources on roadways, excluding highways, in the northeastern part of San Francisco County, California. Modeled concentrations are based on the STREET Gaussian dispersion model developed for urban environments (Johnson et al. 1973).

An important outgrowth of the involvement of SFDPH in EIAs such as the rezoning proposals has been the development of a number of new methods to allow better prediction of health effects across impacted local populations. These methods have been reported elsewhere and include *a*) using an established-traffic noise model, calibrated with available citywide traffic and noise data, to predict area-level variation of population noise exposure and related health risks (Seto et al. 2007); *b*) employing a Gaussian dispersion model to predict air pollutant levels and pollutant-related health effects (SFDPH 2007) (see map in Figure 1 illustrating modeled concentration of PM_2.5_ from traffic sources in northeastern San Francisco County); and *c*) developing a multivariate regression model to predict the impact of rezoning plans on vehicle-pedestrian collisions at the level of the census tract based on transportation network data, proposed land uses, and demographics (Wier et al. 2007).

Another important consequence of this work has been the establishment of day-to-day dialogue between the SFDCP and SFDPH. Because the issues analyzed in the Eastern Neighborhoods were relevant to development throughout San Francisco, the SFDPH and SFDCP are now working to codify some of the mitigation measures as new citywide regulations for planning. SFDCP is also routinely requesting the SFDPH to conduct noise and air quality analyses for other projects.

### Alaska’s north slope: tribes demand an integrated HIA/EIA

The Alaska Inter-Tribal Council (AITC), in cooperation the North Slope Borough (NSB), recently successfully advocated for the inclusion of HIA-based analyses in several federal EISs for North Slope oil and gas development (Wernham 2007). This is the first HIA formally integrated into a federal EIA reported in the United States (Dannenberg et al. 2008). This project was initiated by the affected communities in partnership with one of the authors (A.W.) in response to long-standing community concerns regarding a range of health related impacts experienced by the Inupiat communities in the North Slope region, and evolved through collaboration between community stakeholder groups, public health professionals, and regulatory agencies.

Inupiat communities had raised health concerns related to oil and gas development and its related impacts in public testimony for many years, but most of these concerns were not well addressed in previous NEPA documents. Examples of issues raised by stakeholders include *a*) contaminant-based problems, such as the risk of cancer from consuming tainted fish and game and increases in asthma from exposure to gas flaring; *b*) nutritional impacts, including a shift away from a subsistence diet to store-bought foods accompanied by a rapid increase in diabetes and related metabolic disorders; and *c*) social pathology, including epidemic suicide and domestic violence (rates of which are now among the highest in the United States) and alcohol and drug abuse, attributed in part to the intense sociocultural stresses placed on these small communities by nearby industrial activities.

Working with A.W., the AITC and NSB approached the federal regulatory agencies—the Bureau of Land Management (BLM) and the Minerals Management Service (MMS)—and presented the arguments that *a*) NEPA analyses on prior North Slope development had consistently failed to address public testimony on health concerns; *b*) health impacts fall within the scope of impacts required by NEPA; *c*) public health data are readily available to inform such an analysis; and *d*) HIA provides an appropriate methodology. A dialogue ensued between tribal representatives, BLM and MMS management, and solicitors for the Interior Department. Ultimately, both the BLM and MMS accepted the fundamental premise that health should be included in an EIS, acknowledged that they lacked staff expertise to accomplish this, and invited A.W. to draft appropriate EIS subsections for three active NEPA processes.

Two EISs were already under way. For these, brief subsections on health were drafted and submitted as formal comments during the draft EIS comment period. These rapid HIAs were integrated into the environmental justice chapters in the EISs (MMS 2007a, 2007b). The methodology involved a review of public testimony from the scoping and draft EIS phases and prior related projects, a literature review, and a descriptive analysis of the potential linkages between the environmental disturbances predicted in the draft EIS and health outcomes. Because these HIAs addressed oil and gas leasing programs several stages removed from actual development, and because they were completed late in the EIS process, new mitigation measures were not considered. MMS, however, made a commitment to pursue effective strategies for mitigating impacts to human health in cooperation with the tribes, the NSB, and the AITC and other state and federal agencies (MMS 2007b).

For the third EIS—a supplemental EIS for oil leasing in the National Petroleum Reserve, Alaska (NPR-A)—the NSB became a cooperating agency at the outset of the NEPA process, and the NSB and AITC worked with BLM scientists to draft a fully integrated HIA including new health-focused mitigation measures (detailed in Table 1) (BLM 2007). This HIA was vetted with agency management in Washington, DC, subjected to internal agency reviews, and ultimately included in the EIS with virtually no modification.

The most challenging issue encountered related to the authority of the BLM to regulate based on public health concerns. The BLM operates under the Federal Lands Policy and Management Act (FLPMA), which confers broad authority for land management decisions, but says little about health (FLPMA 1976). Consequently, although evaluating health impacts clearly falls under NEPA responsibilities of the BLM, in some instances BLM felt that it lacked authority under FLPMA to create new regulations based only on health concerns. BLM agreed to include certain measures that clearly fell within its authority (Table 1). Additionally, BLM drafted a new measure that, for specific development proposals in the region, would require developers to work directly with the appropriate health agencies and impacted communities to use HIA to identify potential health impacts and implement new health-based mitigation. Finally, BLM included an appendix to the EIS outlining successful examples of broader mitigation measures from international resource development near indigenous populations.

This work has sparked interest from tribes, state and federal regulatory agencies, and health agencies across Alaska. A tribal health organization recently decided to become a cooperating agency on a U.S. Environmental Protection Agency (EPA)–led mining EIS. Tribal groups, state and tribal health organizations, the Agency for Toxic Substance and Disease Registry, the U.S. EPA, and mining industry executives recently attended a training on HIA led by AITC and the National Center for Environmental Health of the U.S. Centers for Disease Control and Prevention. The U.S. EPA is now considering including a more rigorous and comprehensive approach to health impact analysis for several other anticipated mining EISs. Recently, a multinational oil corporation expressed interest in supporting and building on local HIA efforts in Alaska as a part of its planning efforts for expanded offshore development.

## Lessons Learned

The practice of integrating HIA into EIA is in its infancy in the United States; still, the cases described above suggest that this approach can effectively promote the consideration of health impacts, health determinants, and the needs of vulnerable populations by policy makers in a broad spectrum of activity subject to EIA. Below, the authors discuss several lessons which may help inform similar efforts elsewhere in the United States.

## EIA Requirements Support a Broad Consideration of Health Effects

The case examples suggest two overarching conclusions. First, despite the codified, litigious, and rigid procedures commonly used in EIA, lead agencies will often accept well-reasoned, scientifically grounded public health arguments as justification to expand the scope of an EIA. Second, the scope of health issues that can be addressed through EIA is surprisingly broad, including concerns as diverse as traffic injuries, social cohesion, traditional subsistence diets, social problems such as domestic violence, and psychological problems such as stress from displacement.

These findings can be explained by several observations. First, statutory requirements specific to health in EIA, coupled with the legal mandate for agencies to consider and respond to substantive public input, create a powerful legal platform from which to advocate for health analysis and related mitigation. Second, the routine inclusion of social, economic, and broad-based environmental impact analyses in EIA naturally supports a broad perspective on health effects. Finally, the growing strength of empiric evidence linking social, environmental, and economic conditions to health and health disparities supports forecasting how changes in societal conditions affect health outcomes.

## Integrating Health in EIA Can Impact Public Policy

The case examples also illustrate that the integrated HIA/EIA can result in new policies, regulatory measures, or project designs that protect and promote health. In the case of Trinity Plaza, for example, documenting the potential impacts of displacement on health through the EIA process led the developer to modify the project design to include affordable housing units for existing tenants, mitigating the impact in advance of the EIA. The analysis of roadway air quality impacts in the Eastern Neighborhoods EIA is currently being translated into citywide planning and zoning regulations. In Alaska, integrated HIA/EIA led to the proposed adoption of new regulatory measures to monitor environmental and health indicators and to require HIA and site-specific mitigation for future development proposals. It also initiated a multilateral policy discussion regarding how to promote long-term socioeconomic stability and community well-being.

The power of this approach is being recognized by diverse community and social justice groups. In Oakland, California, for example, tenants’ advocates familiar with the San Francisco experience used public health evidence to articulate how a policy to facilitate the conversion of apartments to condominiums might lead to increased traffic, crowding, poor sanitation, and homelessness, thus requiring an EIA (Perlmutter 2006). On this basis, advocates successfully argued for the city council to send the condo conversion policy back for further study and revision. In Alaska, tribes are now successfully bringing the issue of health into the regulatory process for a number of large industrial proposals.

## Collaboration with Affected Communities Is Essential

In each of the case examples, it was the combination of vigorous public testimony with public health expertise that finally led to the inclusion of health concerns in the EIA process. In both cases, communities had testified to a range of health-related concerns without a substantive regulatory response. In Alaska, communities had raised concerns regarding the impacts of oil development on cultural stability and health in testimony over many years on multiple EIAs. In San Francisco, concerns about the effects of new residential development on community stability and economic livelihoods preceded SFDPH involvement in the EIA process by several years.

Public testimony on EIAs frequently reflects a holistic perspective that naturally links diverse issues as housing affordability, displacement, cultural change, noise pollution, and political and social control with community health and well-being. In turn, health professionals can make a strong case for the importance of such impacts by citing data on the determinants of health, thus validating and strengthening the case for fully considering such issues in an EIA.

Conversely, agencies may disregard the input of health experts unless it is presented directly on behalf or in cooperation with an affected stakeholder community. In 2006, a team of environmental health students and faculty at the University of California at Berkeley (including author R.B.), the UC Berkeley Health Impact Group (UCBHIG), conducted an HIA on the proposed Oak to Ninth Development Project—a new neighborhood with 3,100 new residences and 200,000 square feet of commercial space on 64 acres of publicly owned waterfront land on the Oakland Estuary (Bhatia et al. 2006). The analysis focused specifically on health impacts raised in public testimony that were not adequately addressed in the final EIA of the project (CEDA 2006). Although the HIA occurred late in the regulatory process, it included significant qualitative and quantitative conclusions regarding vehicle–pedestrian collisions resulting from project-generated traffic; noise and air pollutant impacts to future residents; the loss of public land for open space; displacement of low-income households, barriers to waterfront access; and safety of walking or biking to and from the development. Under CEQA, the City of Oakland had an obligation to consider additional facts in determining the adequacy of its EIA. Yet, despite direct and public communication with the planning director, the Oakland Planning Commission, and the Oakland City Council, the HIA had little influence on the EIA, the design of the project, or its subsequent regulatory decision making.

The limitations in this case most likely derived from the lack of standing of the HIA team in the EIA process. As an independent, academic group, the team was neither a cooperating agency nor was it directly collaborating with impacted communities. Although in private some elected officials acknowledged the validity of the HIA, elected officials appeared unwilling to challenge the judgments of the planning director (who had determined the EIA to be adequate) or the strong political support for the project from its many proponents. In this case, a stronger alliance between impacted communities and the HIA team would have given the health issue a stronger standing in the EIA process. Subsequent HIAs conducted by the UCBHIG have involved close partnerships with community stakeholders, with explicit roles for analysis, communication, and advocacy.

## Predictive Judgments in HIA, as EIA, Require Appropriate Standards of Evidence

Compared with empirical public health research, neither EIA nor the policy and planning decisions it informs have rigorous standards for evidence. Such decisions are made at the pace required by political or economic priorities and based on available evidence and professional opinion. Given the methodologic challenges involved in predicting health outcomes, public health professionals must balance the risk of making recommendations based on flawed analyses against the risk that readily preventable adverse health outcomes will not be recognized at all (Dannenberg et al. 2008; Parry and Stevens 2001). By its nature, impact assessment involves reasoned judgments in the setting of multiple assumptions, uncertainties, and often incomplete baseline data (Veerman et al. 2007). In this context, there are two central challenges involved in integrating HIA and EIA. First, standards of evidence must reflect an appropriate balance of rigor and practicality, such that public health can provide beneficial input at the pace required by the EIA process. Second, HIA practitioners must develop an appropriate set of analytical tools.

Causal certainty and quantitative precision are unrealistic and unnecessary standards for EIA. When there is insufficient information to make an important judgment, NEPA requires that either *a*) an agency collect the data only if it can be obtained and the costs are not exorbitant, or *b*) if the information cannot be obtained or costs are too great, the agency rely on accepted theoretical approaches and the assessment is “not based on pure conjecture and is within the rule of reason” (CEQ 1978 §1508.22). Expert opinion has been found repeatedly to constitute a valid basis for EIA conclusions (see, e.g., Greenpeace Action v. Franklin 1992).

The optimal mix of rigor and expediency depends on the decision context. In the case of Trinity Plaza, city officials were initially skeptical about the health effects of displacement, particularly those on mental health and those mediated through impacts on social cohesion. Yet the SFDPH was able to produce compelling data linking affordable housing and displacement to health outcomes, and ultimately, SFDCP officials accepted this central premise without imposing requirements for additional modeling.

Regardless of the scientific validity of HIA conclusions, HIA practitioners should expect some skepticism regarding complex health arguments that involve environmental, social, and behavior pathways. In Alaska, the original skepticism of NEPA analysts and regulators appeared to be due to a basic lack of familiarity with public health principles, such as the links between diet and diabetes or income and general health status, and was resolved through ongoing discussions in which the cooperating agencies openly explored and debated public health principles and evidence.

## Practitioners Need Analytic Tools for HIA

New analytical tools are needed to translate the impacts presented in an EIA into valid health impact predictions. The development of these tools is facilitated by the interdisciplinary approach offered by EIA. For example, forecasting the impact of road traffic on respiratory disease in the Eastern Neighborhood case involved estimating the effect of the land use plans on vehicle flows using existing traffic models and observed traffic counts; the effect of vehicle flows on local air pollutant concentrations using available atmospheric dispersion models; and finally, the effect of pollutant exposure on respiratory disease using dose–response functions from epidemiologic studies.

On the other hand, quantitative forecasting methods such as the SFDPH pedestrian injury collision model are not necessarily more effective than nonquantitative approaches for achieving policy change. Quantitative conclusions may focus debate on the validity of the analytic technique and divert attention from reasonable strategies to address obvious health concerns. For example, despite the rigorous process involved in creating and validating the pedestrian injury model, the SFDCP criticized the methods as unproven. Ultimately, even the most rigorous quantitative impact assessment methods involve assumptions regarding the complex and multilayered inputs of various social, economic, and environmental factors—assumptions that can limit both their acceptance and their validity.

Adaptive management is an approach that recognizes the inherent uncertainty of prospective impact estimates. Adaptive management applies an iterative process in which initial predictions are used to make a management plan, and outcome monitoring is relied on both to adjust the outcome predictions and to modify management strategies (Murray and Marmorek 2003; Steinemann 2000). EIA has been specifically criticized for the lack of this sort of prospective outcome monitoring (Canter and Clark 1997; Karkkainen 2002). In Alaska, several of the proposed health mitigation measures relied on adaptive management, through creating requirements for baseline and ongoing monitoring of health outcomes and specifying a mechanism through which BLM could alter its management requirements based on the monitoring outcomes.

## A Cooperative Interdisciplinary Practice Can Evolve

Despite the statutory support for including health in EIA, the often contentious and adversarial atmosphere surrounding EIA poses a potential barrier to the addition of health issues. Project proponents may resist health analysis because of fear that such analysis is being motivated primarily by opposition to a project and the desire to avoid confrontation, and potential legal challenges may limit the interest of regulators (Cole et al. 2004; Steinemann 2000).

In our experience, however, establishing a mutually respectful interdisciplinary collaboration can mitigate such barriers. In the Eastern Neighborhoods example, SFDPH responded to the concerns of the SFDCP about methodologic consistency by developing analytic methods for noise and air quality impacts and suggesting citywide significance standards. Consequently, the SFDCP accepted much of the subsequent analysis without argument, which shifted the focus of discussions to the feasibility of potential mitigations and design alternatives. In Alaska, after a legal review by agency solicitors, the federal regulatory agencies acknowledged that evidence-based public health information presented by affected communities could not be ignored. In the cooperative relationship that developed, NEPA analysts greeted the additional health information with enthusiasm, commenting that it improved the EIA and helped to give context to other aspects of the analysis, focus the process on the needs of the stakeholders, and reduce the acrimony often present between the agencies and local communities.

Industry proponents should also be viewed as important potential collaborators in integrated HIA/EIA. Increasingly, internal corporate good-neighbor policies include requirements for both EIA and HIA and for comprehensive environment, health, and safety management plans to mitigate identified impacts (International Petroleum Industry Environmental Conservation Association 2005; Sakhalin Energy Investment Company 2006). Directives of the International Finance Corporation (2007) now contain explicit standards and guidance addressing human health. Power imbalances between small communities and large developers can compromise the efficacy of voluntary, corporate impact assessment and mitigation plans. But the use of community-driven, integrated HIA/EIA may offer local communities substantially more leverage in negotiating reasonable agreements. In Oakland, stakeholders, supported by the nonprofit Human Impact Partners (2008), have begun to develop a community-based practice of HIA that involves developers and that informs the city’s EIA process. In the Alaskan case example, BLM felt that it had limited regulatory authority to address several of the specific health concerns and instead suggested measures that would encourage developers to work directly with the impacted community and health authorities to develop health-focused mitigation. Since publication of the Northeast NPR-A draft EIA (Table 1), one multinational oil developer has approached the NSB to discuss collaboration on HIA and sustainable development planning.

## Conclusion: A Vision and Recommendations for Integrated Practice

In the environmental sector, policy debates are commonly framed as conflicts between environmental preservation and the economic well-being of communities. This perspective ignores the interdependence of human health and the integrity of the natural environment, as well as the complex social, economic, and health effects of environmental management decisions. In the health sector, on the other hand, objectives such as those outlined in Healthy People 2010 center on promoting healthy communities and eliminating health disparities (U.S. Department of Health and Human Services 1999). Population health goals can only be achieved through a truly cross-sectoral approach that engages every agency that makes decisions that impact social, economic, and environmental conditions. We believe that EIA presents an opportunity for the field of public health to participate in a cross-sectoral approach with influence on the planning, evaluation, and execution of a wide range of activities that fundamentally shape living conditions in communities across the United States.

These case examples demonstrate that a legal framework supporting the inclusion of health in EIA in the United States exists at the federal level and likely in many states as well. Public health agencies, academic institutions, and professionals should view EIA as a potentially effective tool to integrate health objectives into a wide range of policy decisions. Health analysis under NEPA can be accomplished by a public health agency, a university, or a private public health consultant working in collaboration with the impacted community and the lead agency. Realizing a vision of an integrated health and environmental analysis would enable a powerful policy lever for population health and health equity. To achieve these goals, the authors offer the following recommendations:

### Engagement with local EIA and lead agencies

Public health agencies and academic institutions should familiarize themselves with regional EIA activities, and participate, either through HIA or simply through providing comments, where either public testimony or obvious public health concerns indicate a need.

### Engagement with impacted communities

Health agencies should familiarize themselves with community concerns regarding active EIAs and partner with the community to explore how public health data and expertise might be used to inform these concerns.

### Capacity and workforce needs

Effective participation in the EIA process will require public health staff with time and at least a basic familiarity with EIA and HIA. Only one university graduate school course on HIA exists in the United States. Schools of public health and continuing education courses should consider offering HIA training as part of a core skill set for public health professionals.

### Funding HIA

Some involvement in EIA may be feasible within existing budgets of public health agencies through the reprioritization and training of existing staff positions. Agencies without such flexibility should evaluate potential alternative funding mechanisms for EIA participation, including direct payments by developers (required in some EIAs); funding from lead agencies for cooperating agency work (rare); regulatory agency grant programs (a number of agencies have programs, such as U.S. EPA environmental justice grants, that could be applicable); and private grantors. Participation in EIA may prove to be a cost- and time-effective health intervention in the long run.

### Guidance for health analysis

Health and environmental regulatory agencies should advocate for formal federal guidance on incorporating health in EIA, building on the examples of Australia, Canada, and the International Finance Corporation. It is worth noting that guidance for social impact assessment and environmental justice contributed to institutionalizing these issues as routine considerations within EIA (Bass 1998; Burdge 1988)

### Evaluation criteria and monitoring

Evaluation criteria have been proposed for HIA, but HIA practice has been subject to limited evaluation (Parry and Kemm 2005). Issues important for HIA/EIA evaluation include analytic validity, issue relevance, public involvement, and impacts on decisions as well as decision makers and decision-making practices. HIA/EIA integration also offers the opportunity to institute adaptive management or mitigation measures that require ongoing monitoring of both health outcomes and environmental factors known to affect health, an effort that will contribute to the efficacy and accuracy of HIA methods.

### Collaboration with other HIA and EIA proponents

NEPA called for an interdisciplinary analysis on all environmental issues important to people (CEQ 1978 §1502.6). Open collaboration and discussion between all professionals interested in HIA/EIA will facilitate the success of individual efforts.

## Figures and Tables

**Table 1:** Key elements of HIAs integrated within EIAs in California (San Francisco) and Alaska, United States.

Policy program or project Description Location, year	Organization conducting HIA	Integrated HIA /EIA approach and research methods	Key findings	Regulatory outcomes
Trinity Plaza Redevelopment Proposal to demolish and redevelop rent-controlled housing as private condominiums San Francisco, CA, 2003	SFDPH	Desktop HIA: scope based on document review and public testimony; appraisal through expert judgment supported by empirical evidence, local secondary data, and focus group findings; findings provided as comments on scope of EIR.	Project would result in displacement, housing cost burdens, relocation, or substandard housing for evicted tenants; mitigation suggested to provide replacement housing for existing tenants.	Decision makers required the project proponent to provide replacement housing for existing residents or analyze displacement impacts in an EIR.
Eastern Neighborhoods Rezoning and Area Plans Rezoning of four industrial and mixed-use neighborhoods for greater residential use San Francisco, CA, 2007	SFDPH	Qualitative and quantitative analysis integrated within EIA. Scope: roadway air pollutants; land use—noise conflicts, and pedestrian safety. Appraisal based on expert judgment, regulatory experience, noise and air quality exposure analysis, pedestrian collision modeling, quantitative health hazard assessment, and research on mitigation strategies.	Significant impacts on mortality and respiratory disease due to roadway–residential use proximity; impacts on noise exposure and business displacement due to industrial–residential use conflicts; impacts on pedestrian collisions due to roadway–residential use conflicts. Suggested mitigations included building ventilation and filtration; noise assessment and acoustical controls; traffic calming; pedestrian safety engineering countermeasures; and traffic demand reduction.	The draft EIR included health impacts analysis of roadway pollution, noise, and pedestrian collisions, related findings of significance. Mitigation measures included requirements for project level noise and air quality assessments and protective building design.
Chukchi Sea Planning Area Oil and Gas Lease Sale 193 Sale of gas lease Anchorage, AK, 2007	AITC and North Slope Borough	Desktop HIA including description of logic pathways, supported by public health data and public testimony on related EISs.	Similar impacts in each of the three HIAs: Displacement of subsistence animals and hunters, leading to dietary change, and increased risk of diabetes, obesity, hypertension, cardiovascular disease, and food insecurity. Disruption of environment coupled with marked influx of oil and gas workers leading to social strain, cultural change, and the potential for increased access to drugs and alcohol, with the resultant increased risk of social pathology(domestic violence, suicide, drug and alcohol problems) and injury. Potential infectious disease transmission between oil camps and villages.	Not addressed.
Outer Continental Shelf Oil and Gas Leasing Program: 2007–2012 Washington, DC, 2007	AITC and North Slope Borough	Desktop HIA including description of logic pathways, supported by public health data and public testimony on related EISs.		Agreement to address new health-focused mitigation at the lease-sale stage.
Northeast NPR-A Supplemental EIS Anchorage, AK, 2007	AITC and North Slope Borough	Integrated HIA/EIS: combination of public meetings, literature review, and scientific meetings among EIS team and public health experts used to delineate impact pathways and project qualitative outcomes.		New mitigation measures considered in EIS, including: Requirement that industry must identify and mitigate any possible health impacts for all development plans in the region. Monitoring of subsistence harvest, with restrictions in development activity if reductions attributable to development occur. Monitoring of health indicators, with potential modifications in development operations if adverse outcomes occur. Monitoring of contaminants in environment and subsistence foods. Employee orientation to include infection control and drug/alcohol policy information. Creation of a scientific review panel to monitor and mitigate health impacts in the case of a large oil spill.
